# A rapid assay for assessing bacterial effects on *Arabidopsis* thermotolerance

**DOI:** 10.1186/s13007-023-01022-0

**Published:** 2023-06-29

**Authors:** Jun Hyung Lee, Leah H. Burdick, Bryan Piatkowski, Alyssa A. Carrell, Mitchel J. Doktycz, Dale A. Pelletier, David J. Weston

**Affiliations:** Biosciences Division, Oak Ridge National Laboratory, 1 Bethel Valley Rd Bldg. 1507, Rm. 214, Oak Ridge, TN 37831 USA

**Keywords:** Heat stress, Thermotolerance, Phenotyping, Chlorophyll content, High-throughput, Rapid assay, Plant-microbe interactions

## Abstract

**Background:**

The role of beneficial microbes in mitigating plant abiotic stress has received considerable attention. However, the lack of a reproducible and relatively high-throughput screen for microbial contributions to plant thermotolerance has greatly limited progress in this area, this slows the discovery of novel beneficial isolates and the processes by which they operate.

**Results:**

We designed a rapid phenotyping method to assess the effects of bacteria on plant host thermotolerance. After testing multiple growth conditions, a hydroponic system was selected and used to optimize an *Arabidopsis* heat shock regime and phenotypic evaluation. *Arabidopsis* seedlings germinated on a PTFE mesh disc were floated onto a 6-well plate containing liquid MS media, then subjected to heat shock at 45 °C for various duration. To characterize phenotype, plants were harvested after four days of recovery to measure chlorophyll content. The method was extended to include bacterial isolates and to quantify bacterial contributions to host plant thermotolerance. As an exemplar, the method was used to screen 25 strains of the plant growth promoting *Variovorax* spp. for enhanced plant thermotolerance. A follow-up study demonstrated the reproducibility of this assay and led to the discovery of a novel beneficial interaction.

**Conclusions:**

This method enables rapid screening of individual bacterial strains for beneficial effects on host plant thermotolerance. The throughput and reproducibility of the system is ideal for testing many genetic variants of *Arabidopsis* and bacterial strains.

## Background/Introduction

In the past hundred years, the average global temperature has increased by 1.1 °C and is projected to continue increasing at a rate of 0.2 °C per decade [1]. In addition to increasing average temperature, global climate change has been linked to more frequent and severe extreme temperature events such as heat waves which have increased in frequency, intensity, and duration [2, 3]. Heat stress is a major environmental factor that negatively affects plant growth and productivity, and ultimately threatens world food security. For instance, extreme heat events have reduced global cereal production by 9.1% from 1964 to 2007, mainly due to grain yield deficit [4]. Furthermore, crop models also predict that each degree Celsius increase in global average temperature would reduce global yields of wheat by 6%, rice by 3.2%, maize by 7.4%, and soybean by 3.1% [5, 6].

To cope with heat stress, plants are able to develop tolerance upon exposure to increased but non-damaging temperature, known as thermo-priming, which can allow them to survive subsequent heat stress [7]. The involvement of many genes including *HSFs* (heat shock transcription factors) and *HSPs* (heat shock proteins) in thermo-priming has been extensively studied [8]. Distinct from this acquired thermotolerance, plants have the innate ability to survive under heat stress (i.e., basal thermotolerance) which depends on plant species and genotype. Using thermotolerant germplasm and/or well-studied genes in heat response, multiple programs are currently underway to improve plant thermotolerance mainly through traditional breeding or bio-design engineering efforts [9, 10]. However, an emerging approach to enhance thermotolerance is through plant associations with beneficial microbes. Studies have investigated the bacterial association on plant growth, commonly termed plant growth-promoting bacteria (PGPB), under heat stress. Several examples of PGPB enhanced plant thermotolerance have been reported using wheat, sorghum, soybean, and tomato [11–14]. Recent studies show that PGPB alter host metabolism which can reduce heat-induced membrane injury, increase HSP levels, and alter chromatin modification *via* heat stress memory loci [12, 15, 16]. However, plant - PGPB interactions are highly species- and genotype-specific [17, 18], therefore the mechanism underlying the bacterial-provided thermal benefits is largely unknown.

Assays for plant thermotolerance have been developed to study plant heat acclimation and screen mutant lines [7, 19]. Critical assay components to consider when designing a high-throughput reproducible experimental protocol to assess plant thermotolerance are the heat stress regime and plant phenotyping. Heat stress regime can be determined based on the intensity and duration, of which the impact largely depends on plant species, culture system, physiological state, and developmental stage [20, 21]. Various indicators have been used to distinguish plant phenotypes under heat stress, including hypocotyl elongation, survival rate, biomass, ion leakage, chlorophyll content and fluorescence, gene expression, and seed germination [16, 19, 22–26]. However, no standard approach has been developed to explore bacterial effects on plant host thermotolerance. A reliable, high-throughput assay to quantify such beneficial effects could help to identify the molecular genetics governing thermotolerance from the perspective of both the bacteria and their plant hosts.

In the present study, we developed a rapid assay to assess bacterial effects on *Arabidopsis* thermotolerance. We introduced a hydroponic-based culture system to simplify the inoculation process and provide consistent heat stress. The basal thermotolerance of seedlings was quantified by measuring chlorophyll content under different heat durations. We utilized this assay to screen *Variovorax* strains for the ability to provide benefits to plants to heat stress. We subsequently performed additional analyses on a promising strain to confirm the bacterial provided thermotolerance. This assay is not only amenable to high-throughput screening of individual bacterial strains but can also scale to include multiple *Arabidopsis* genetic variants and microbial consortia.

## Results and discussion

### Establishment of culture system and phenotypic evaluation for heat shock bioassay

**Fig. 1:**
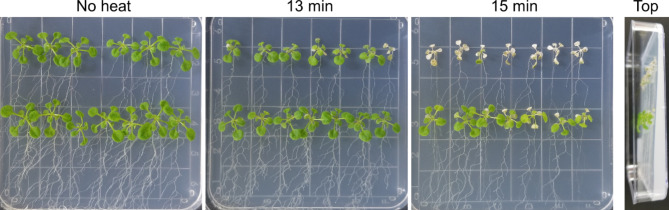
**MS agar plate-based heat shock bioassay. ***Arabidopsis* seedlings vertically grown on MS agar plate were subjected to heat shock at 45 °C for various durations. The medium on the upper part of the plate dried more quickly and became thinner, resulting in more damage from heat shock compared to plants on lower positions

The basal level of plant thermotolerance depends on multiple factors including plant developmental stage and physiological state. Because growth conditions can influence plant development, we tested multiple systems. First, we tested a vertical agar approach where *Arabidopsis* seedlings were placed on MS agar plates in a vertical position. After two weeks of growth, seedlings were subjected to heat shock at 45 °C, and then transferred to normal growth condition at 22 °C for recovery for 4 days before performing phenotypic assays. Results from this approach were varied and highly inconsistent depending on the seedling position within the plate (Fig. 1). Plants on the top row showed higher percentage of bleached leaves compared to plants on the middle row: 30.2 ± 22.9% (top) and 0% (bottom) with 13 min heat shock (*P* < 0.001); 97.6 ± 6.3% (top) and 29.6 ± 16.0% (bottom) with 15 min heat shock (*P* < 0.001). It is likely that these inconsistencies are due to variation in agar medium thickness and water status across the vertical plate as the top portion of the gel dried more quickly than that of the bottom area. The difference in thickness likely resulted in more damage from heat shock to the plants grown on the thinner part relative to those on the thicker part, leading to inconsistency in evaluation of thermotolerance. Even when seeds were placed on the same line of each plate, other factors like positional effect in the growth chamber which affects air flow made it difficult to maintain a consistent amount of medium per plate. Similar concerns with agar plate-based thermotolerance assays prone to inconsistent results, both between and within plates, have been previously mentioned unless proper caution is used when performing the assay [7, 21].

**Fig. 2:**
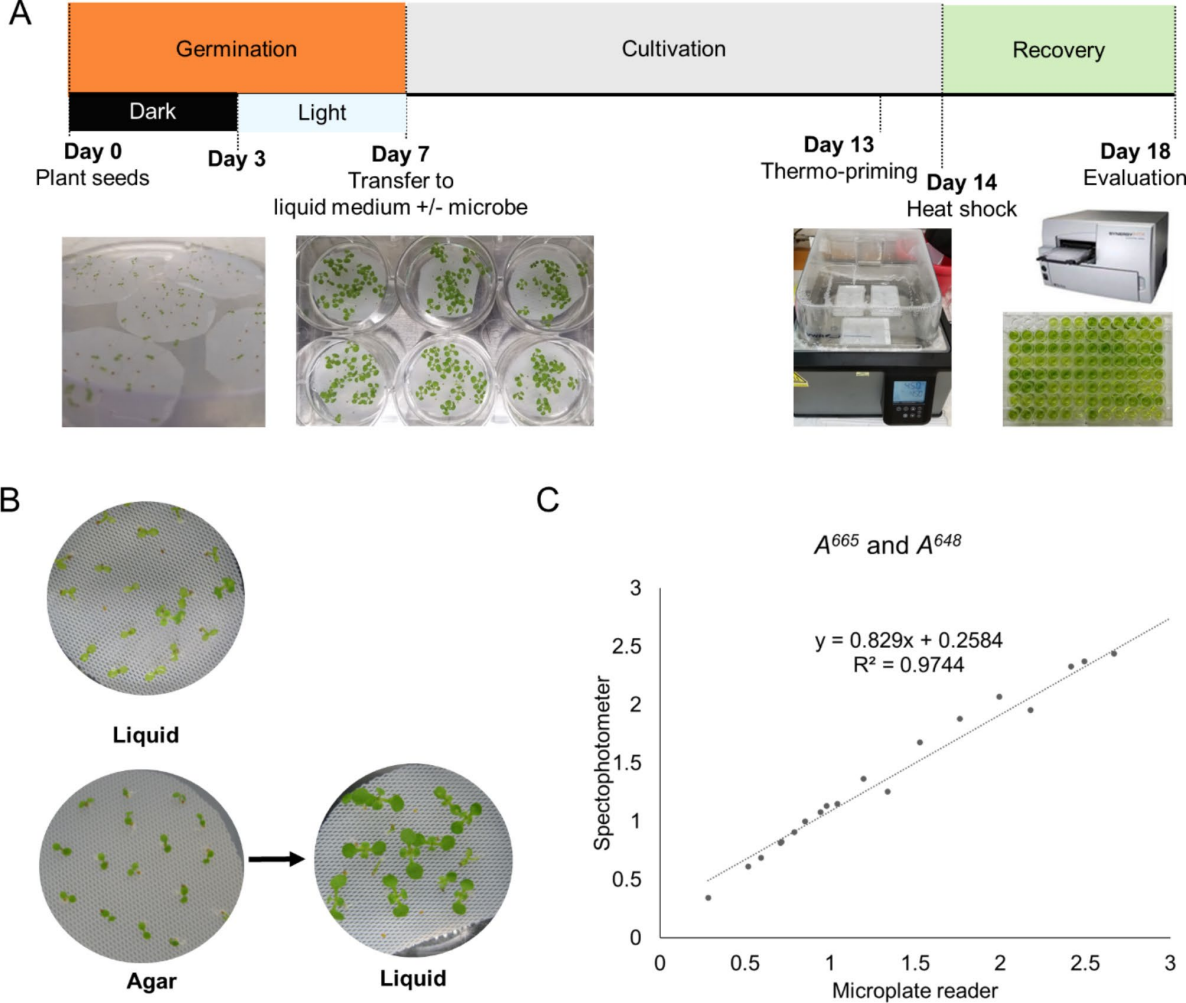
** A rapid bioassay to evaluate plant thermotolerance.** (A) Experimental design of heat shock bioassay using hydroponic system. (B) Seedlings germinated directly on the MS liquid medium showed low rates of true leaf development and stopped growth. Instead, seedlings germinated on the MS agar plate then transferred to the MS liquid medium were able to establish well. (C) Linear regression showed high correlation between absorbance at 665 and 648 nm (*A*^*665*^ and *A*^*648*^) measured by the spectrophotometer and microplate reader

To overcome the inconsistent medium effect, we tested a hydroponic culture system based on the method developed by Voges et al. [38] which allows for an even amount of liquid medium before heat shock treatment. Seeds were placed on a PTFE mesh raft and then floated on liquid MS medium. However, the rate of true leaf development was poor when seeds were germinated directly on the liquid medium (Fig. 2B, upper image). When it comes to hydroponic culture for *Arabidopsis*, establishment of the root system is difficult to achieve as young seedlings are sensitive to hypoxic stress [27]. Alternatively, a PTFE mesh raft with seeds was first placed on a solid MS agar plate (7 days) to allow root system establishment before transfer to liquid medium. This approach yielded over 90% of the plants progressing to true leaf development (Fig. 2B, bottom images). After an additional 7 days within the liquid medium, the same volume of fresh medium was replaced in each well providing equal amount of medium to minimize the inconsistent medium effect, and then seedlings were subjected to heat shock at 45 °C followed by a recovery period for 4 days at 22 °C (Fig. 2A).

Total chlorophyll content was measured after the recovery period to estimate the basal level of thermotolerance. Chlorophyll is a crucial plant pigment in the photosynthetic process, and it is highly sensitive to environmental factors such as heat which results in loss of chlorophyll content and eventual decrease in photosynthetic capacity and productivity [28]. Thus, chlorophyll content has been extensively used as a reliable indicator to estimate plant health and productivity [29, 30]. To speed up the measuring process for a high-throughput assay, we used a 96-well plate-based microplate reader rather than a cuvette-based spectrophotometer which is slow as it can only measure one sample at a time. As published equations for estimating chlorophyll content are based on values measured by a standardized 1-cm pathlength spectrophotometer [31, 32], values (*A*^*665*^ and *A*^*648*^) obtained from the microplate reader were corrected by multiplying by 1.6 before being applied to the equation. The estimated chlorophyll content measured by the microplate reader showed a strong linear correlation with the results obtained from a spectrophotometer using 1 mL cuvettes (*R*^*2*^ = 0.9744, Fig. 2C).

**Fig. 3:**
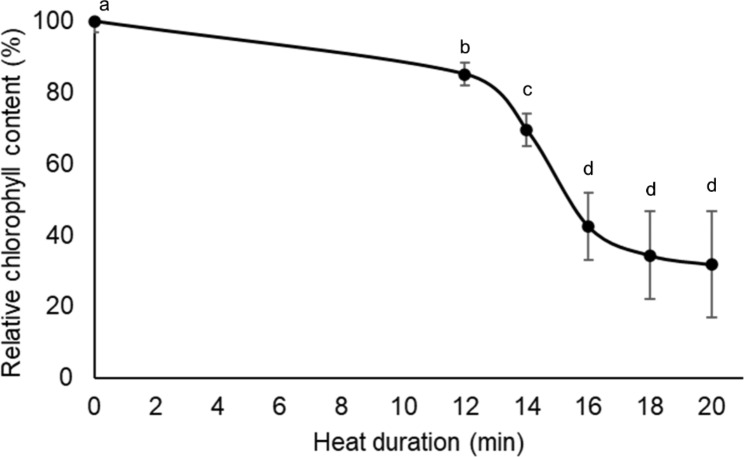
**Assessment of heat shock duration.** Hydroponically grown *Arabidopsis* seedlings were subjected to heat shock at 45 °C for different time periods, then harvested after 4 days of recovery to measure chlorophyll content. Error bars represent the standard deviation (n = 12 wells with ca. 10 seedlings each). Different letters indicate significant differences between treatments (Fisher’s LSD, *P* < 0.05)

The impact of heat shock on plant health can be varied based on the intensity (i.e., temperature) and duration of the shock. In terms of temperature, heat shock at 45 °C has been commonly used for *Arabidopsis* seedlings; however, the length of heat shock was widely different, ranging from 15 min to several hours, depending on the physiological state, developmental stage, tissue types, and the aims of each study [7, 19, 33, 34]. Using the hydroponic culture system and microplate-based chlorophyll measurement method, we assessed the effect of heat shock duration (12–20 min range with 2 min increments at 45 °C). Results indicated that *Arabidopsis* seedlings were sensitive to a short term of heat shock (Fig. 3). Chlorophyll content was gradually reduced as the heat shock duration increased and a sharp drop was observed after 14 min of 45 °C exposure. Silva-Correia et al. [19] also reported a dramatic loss of viability in 7-day-old *Arabidopsis* seedlings (seedling survival rate of over 90% and below 20% with 10 and 15 min of heat shock at 45 °C, respectively), suggesting even small differences in heat shock duration can result in different outcomes. Thus, heat shock duration should be carefully determined for each study. A wide variation in chlorophyll content was observed within the samples treated by heat shock over 16 min, which is due to spurious increases in the fresh weight of bleached/dead seedlings that were soaked thoroughly in liquid medium (Fig. 3). Given these results, we selected 14 min of heat shock for further experiments.

### Screening of bacterial strains for host plant thermotolerance

**Fig. 4:**
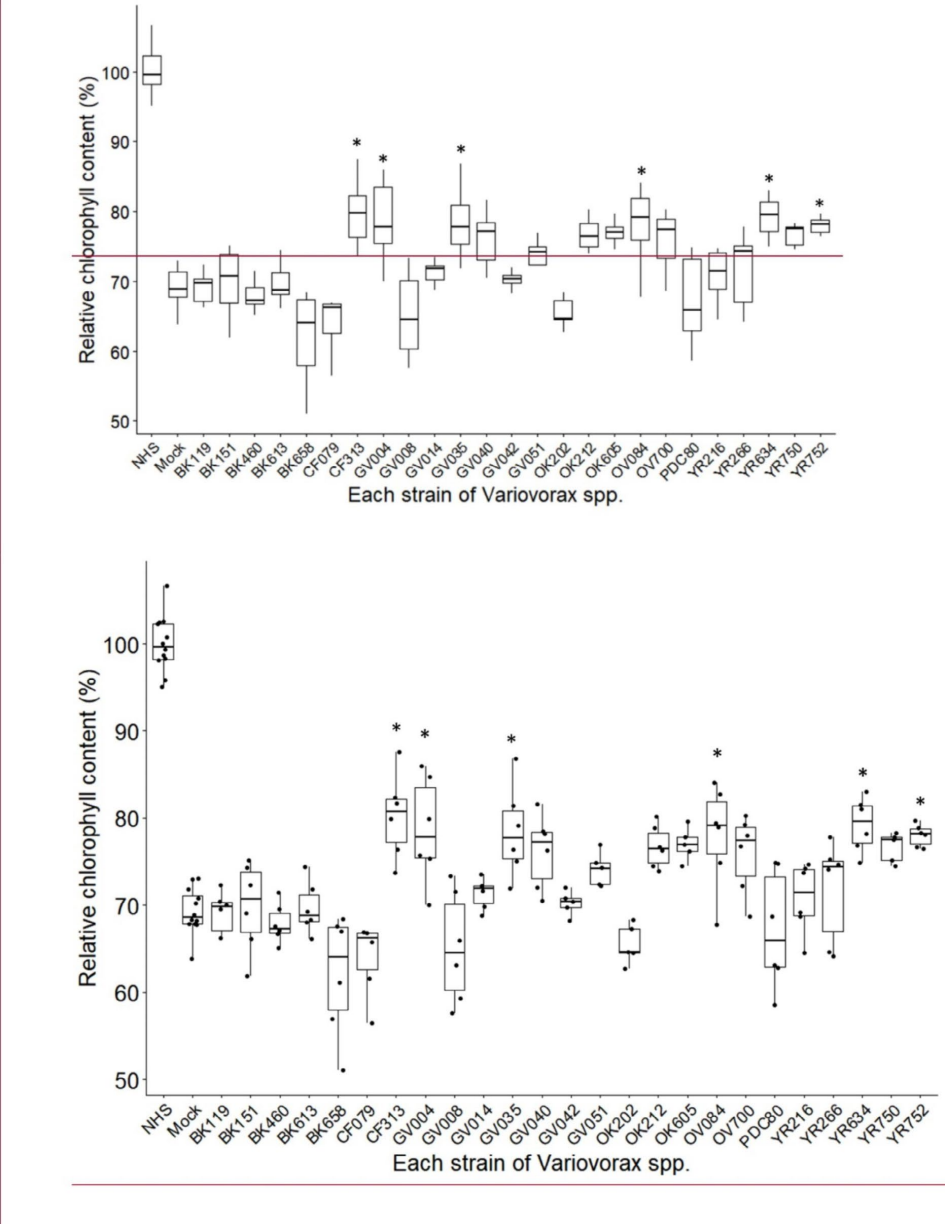
**Assessment of bacterial effects on host plant thermotolerance.** Hydroponically grown *Arabidopsis* seedlings were inoculated with individual strains of *Variovorax* spp. and co-cultured for 7 days prior to heat shock at 45 °C for 14 min. After 4 days of recovery, plants were harvested to measure chlorophyll content. Box plots represent relative chlorophyll content compared to no heat shock (NHS) control plants. Box plots display the 25th – 75th percentiles with the median (horizontal line) (n = 5–6 wells, except NHS and Mock of which n = 12, with ca. 10 seedlings in each well, outliers which lie outside of 1.5 times the interquartile range above the upper quartile and below the lower quartile are removed). Asterisks denote significant differences compared to mock control (Fisher’s LSD, *P* < 0.05)

Twenty-five bacterial strains from the genus *Variovorax* were examined for their ability to enhance host plant thermotolerance. These bacteria are among the core taxa present in the root microbiota of diverse plant species and are known to improve plant root growth [35, 36]. Individual strains of *Variovorax* were added to MS liquid medium to inoculate 7-day-old seedlings and co-cultured for an additional 7 days before heat shock treatment. After 4 days of recovery, plants were harvested, and chlorophyll was extracted. Across all treatments, chlorophyll content was reduced from 21 to 38% compared to the no heat shock (NHS) control (Fig. 4). Among the 25 *Variovorax* treatments, plants inoculated with six strains (CF313, YR634, GV004, GV035, YR752, and OV084) showed significantly higher levels of chlorophyll content compared to the no-bacteria heat shocked plants (Mock), indicating bacterial provided thermotolerance (Fig. 4). To confirm these results, we selected one strain (CF313) for additional experiments.

**Fig. 5:**
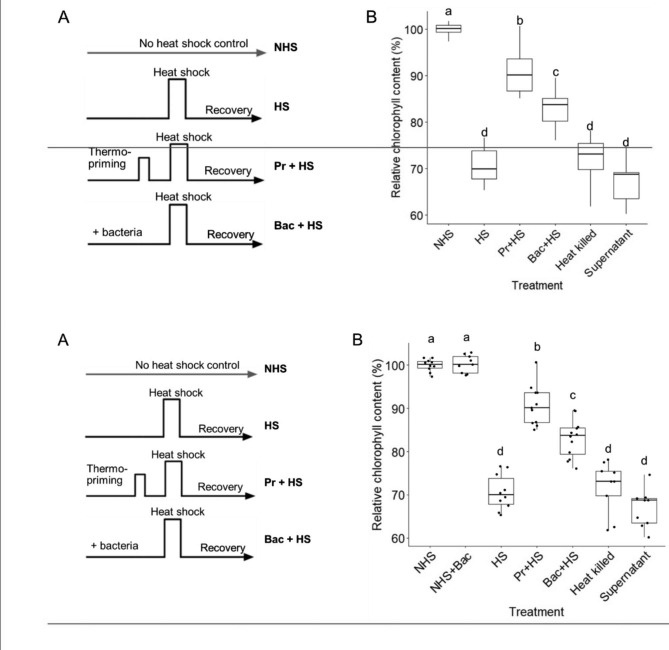
**Confirmation of bacterial provided thermotolerance.** (A) Heat shock treatment regime: NHS, no heat shock control; NHS + Bac, inoculated plants with no heat shock; HS, heat shock at 45 °C; Pr + HS, thermo-priming followed by heat shock at 45 °C; Bac + HS, inoculate plants with bacteria prior to heat shock at 45 °C. (B) Hydroponically grown *Arabidopsis* seedlings were inoculated with live or heat-killed *Variovorax* sp. CF313 and co-cultured for 7 days prior to heat shock at 45 °C for 14 min. The supernatant of the bacteria culture solution was added to test for an indirect effect of bacterial-produced molecules on plant thermotolerance. After 4 days of recovery plants were harvested to measure chlorophyll content. Box plots represent relative chlorophyll content compared to non-heat shocked (NHS) control plants. Box plots display the 25th – 75th percentiles with the median (horizontal line) (n = 9–12 wells with ca. 10 seedlings each). Different letters indicate significant difference between treatments (Fisher’s LSD, *P* < 0.05)

To confirm the effect of *Variovorax* sp. CF313 on enhanced thermotolerance in *Arabidopsis*, we evaluated the chlorophyll content by comparing the no-bacteria heat shocked (HS) plants to the thermo-primed (Pr) plants and to plants inoculated with CF313 (Bac). Thermo-primed plants were exposed to 37 °C for 90 min one day prior to heat shock (Fig. 5a). Compared to NHS control plants, HS plants showed a major reduction in chlorophyll content (− 20.4%) while thermo-primed (Pr + HS) plants showed only 9.3% reduction after heat shock (Fig. 5b). Plants inoculated with CF313 (Bac + HS) exhibited 17% reduction of chlorophyll content which is lower than that of uninoculated plants (HS) but higher than that of thermo-primed plants (Fig. 5b). These results suggest that CF313 enhanced thermotolerance in *Arabidopsis* but to a lesser degree than thermo-priming. Plants that were inoculated with heat killed CF313 or the supernatant of CF313 culture exhibited similar phenotype with HS plants (Fig. 5b), indicating that the thermal benefit conferred by CF313 is from direct contact with living bacteria.

The mechanisms underlying plant benefits from CF313 are not fully elucidated within the current experiment. Plant-microbe interactions are highly complex process which includes phytohormone homeostasis mediated by PGPBs. Under stress conditions plants overproduce ethylene which inhibits plant growth and development [37], therefore lowering the level of ethylene in plants can mitigate abiotic stresses. Various studies have reported that the application of ACC (1-aminocyclopropane-1-carboxylic acid) deaminase-producing PGPBs moderated ethylene metabolism and conferred thermotolerance to plant hosts [14, 38, 39]. *Variovorax* is an ACC deaminase-producing PGPB that can stimulate plant growth under normal growth condition by reducing both the ACC concentration in the leaves and foliar ethylene emission [40]. In this study, we demonstrate that *Variovorax* enhances *Arabidopsis* thermotolerance through yet unknown mechanisms which might be related to ethylene signaling. However, this assay can easily include *Arabidopsis* mutants targeting specific pathways of interest, thereby enabling future efforts to elucidate underlying molecular genetic mechanisms.

## Conclusion

This study provides a robust method for screening *Arabidopsis* thermotolerance in the context of bacterial provided benefits to heat. Our assay enables the rapid evaluation of individual bacterial strains on plant thermotolerance and can also be used to study the emergent effects of bacteria-bacteria interactions within a community. Future studies will focus on comparative genomics using multiple plant genotypes and bacterial strains to elucidate the molecular genetic mechanisms underlying how plants receive thermotolerance benefits from bacteria. In addition, as this assay employs a hydroponic system and liquid inoculum, automation of the whole process from inoculation to phenotyping would be possible.

## Methods

### Plant preparation

Wild-type *Arabidopsis thaliana* (Col-0) seeds were first sterilized in 1 mL of 70% (v/v) ethanol for 1 min, then treated with 1 mL of bleach solution [50% (v/v) bleach/0.05% (v/v) Tween-20] for 5 min with vortexing. Surface-sterilized seeds were rinsed five times with sterile water, re-suspended in sterile water, and then stored at 4°C in the dark for 3 days before planting. For the MS agar plate assay, stratified seeds were planted onto plates containing MS media (4.3 g Murashige & Skoog salts, 0.5 g MES, 6 g agar per liter; pH 5.7; 40 mL per plate), and the plates were placed in a growth chamber vertically for 2 weeks before heat shock treatment. For the hydroponic-based assay, we used autoclaved circular PTFE mesh discs (0.025”x 0.005” opening, McMaster-Carr) following Voges et al. [38] method with a slight modification [41]. About 12 seeds were individually planted in a square grid on each disc. Discs were then placed onto a plate containing MS media and germinated in the dark for 3 days to stimulate hypocotyl elongation which prevent shoots from getting wet. Plates were then moved to the light condition for further growth. One-week-old seedlings on mesh discs were then transferred to 6-well plates (VWR) that contained 3 mL of 1x liquid MS medium in each well. Plants were grown in a growth chamber (Precision™ Plant Growth Chamber, Thermo Scientific) under a 12 h light cycle with 100 µmol photon m^− 2^s^− 1^ at 22 °C.

### Heat shock regime

Two-week-old seedlings were subjected to heat shock at 45 °C by placing the plates onto a temperature-controlled water bath. To minimize other stress factors like light, the water bath was covered with aluminum foil. To provide equal amount of medium per each well, we replaced medium with 2 mL fresh liquid MS medium right before the heat shock treatment. After heat shock treatment, plants were placed back to normal growth condition for recovery for 4 days. For thermo-priming, plants were exposed to non-lethal temperature at 37 °C for 90 min one day prior to heat shock. At the end of the experiments, plants were harvested for phenotyping.

### Bacteria preparation and plant inoculation

Twenty-five *Variovorax* strains isolated from the roots of *Populus* were used in this study [35]. Each strain was streaked on R2A agar plate and cultured at 28 °C for two days. A single bacterial colony from the agar plate was inoculated into 3 mL of R2A broth and cultured at 28 °C with shaking at 250 rpm overnight, spun down, and the pellet was washed 3 times with liquid MS medium. The OD_600_ was determined using a BioTek microplate reader, and then diluted to a final OD_600_ of 0.01 (2.9 × 10^7^ CFU/mL) in liquid MS medium before adding to a 6-well plate (3 mL per well) to inoculate seedlings. To test if the bacterial effects to plant thermotolerance are direct or indirect, heat-killed bacteria and supernatant of bacterial culture filtered through 0.45 μm filter were also used.

### Chlorophyll content measurement

To characterize phenotype, whole rosettes in each mesh disc were harvested together and weighed, then placed in a glass test tube with 2 mL of 100% dimethyl sulphoxide (DMSO) to extract chlorophyll. Sample tubes were incubated at 65 °C for 10 min followed by cooling at room temperature in the dark for 10 min. Once all tissue was discolored, 200 µL of extract was pipetted to a 96-well plate and light absorbance at 665 and 648 nm was measured using a BioTek microplate reader. The absorbance was then converted into 1 cm pathlength-compatible values by multiplying by 1.6 in order to be used in the spectrophotometric equation for estimating chlorophyll content. The concentrations of total chlorophyll (mg/g fresh weight) were calculated using the following equation: total chlorophyll (mg/L) = 7.49ˑ*A*^665^ + 20.34ˑ*A*^648^, multiply 0.002 L, then divide by fresh weight (g) of ca. 10 seedlings in one well [31].

## Declarations

### Data accessibility

The datasets generated and analyzed during the current study are available from the corresponding author on reasonable request.
